# Supplementary material for: Sub-nanosecond heat-based logic, writing and reset in an antiferromagnetic magnetoresistive memory
Source: Photonix. 2025 Nov 4;6(1):46. doi: 10.1186/s43074-025-00207-1 (PMC12586222; doi:10.1186/s43074-025-00207-1)
Supplement: Supplementary file 1 — Supplementary Material 1: Supplementary Note 1. Pulse-multiplier experimental setup (containing Figure S1). Supplementary Note 2. Volume of the switched CuMnAs (containing Figure S2). Supplementary Note 3. Measurement of heat dissipation speed from CuMnAs films to GaP substrate (containing Figure S3). Supplementary Note 4. Dependence of STM dynamics on pulse spacing (containing Figure S4). Supplementary Note 5. Heat-based control of LTM erasing (containing Figures S5 and S6), and Supplementary references. [file 43074_2025_207_MOESM1_ESM.pdf]

Sub-nanosecond heat-based logic, writing and reset in an antiferromagnetic magnetoresistive memory: Supplementary information

M. Surýnek[1], A. Farkaš[2,1], J. Zubáč[2,1], P. Kubaščík[1], K. Olejník[2], F. Křížek[2], L. Nádvorník[1], T. Ostatnický[1], R.P. Campion[3], V. Novák[2], T. Jungwirth[2,3], and P. Němec[1,*]

[1]Faculty of Mathematics and Physics, Charles University, Ke Karlovu 3, 121 16 Prague 2, Czech Republic

[2]Institute of Physics ASCR, v.v.i., Cukrovarnická 10, 162 53 Prague 6, Czech Republic

[3]School of Physics and Astronomy, University of Nottingham, Nottingham NG7 2RD, United Kingdom

CONTENTS

Supplementary Note 1. Pulse-multiplier experimental setup 2

Supplementary Note 2. Volume of the switched CuMnAs 4

Supplementary Note 3. Measurement of heat dissipation speed from CuMnAs films to GaP substrate 6

Supplementary Note 4. Dependence of STM dynamics on pulse spacing 8

Supplementary Note 5. Heat-based control of LTM erasing 10

[*] Electronic mail: petr.nemec@matfyz.cuni.cz

Supplementary Note 1. Pulse-multiplier experimental setup

As described in the main paper, to be compatible with GHz frequencies of conventional electronics, we aimed at experiments with bursts of laser pulses with a mutual time spacing in sub-nanosecond time range. However, standard commercially available femtosecond lasers generate light pulses with repetition rates of only up to ≈ 100 MHz, where the time spacing between these pulses is 10 ns or more. Therefore, we had to construct the pulse-multiplier experimental setup, described in this text and depicted in Fig. S1a, that splits a single 150 fs long laser pulse, generated by our femtosecond laser system with a pulse-on-demand functionality, into its nearly identical copies. A key component in this setup is a doubler stage (DS in Fig. S1a). Here, an incoming laser pulse with a diagonal polarization is split by a polarization beam splitter (PBS) into two pulses, the transmitted horizontally-polarized pulse (blue in Fig. S1a) and the reflected vertically-polarized time-delayed pulse (red in Fig. S1a), which are spatially recombined by the second PBS. Seemingly, this might look as a quite straightforward task. In reality, however, this is a rather challenging achievement due to the non-ideal properties of commercially available PBS. In particular, the plate-shaped PBS distort the spatial profile of transmitted/reflected beams considerably due to the not-normal angle of incidence on their surface, which does not enable the needed perfect spatial overlap of the spatially recombined pulses after the second PBS without performing their Fourier-based spatial filtration. On the other hand, the cube-shaped PBS, where the beam spatial distortion is absent due to the normal angle of incidence, do not have an ideal polarization properties because they are optimized only with respect to the polarization of transmitted light while the polarization-quality of reflected light is much lower and, which is even worse, the polarization plane of these to beams is *not* mutually perpendicular (typically, the deviation from the "expected" angle between the corresponding polarization planes of 90 deg is several degrees). Consequently, after a serial concatenation of several doubler stages this leads to considerable intensity variations between individual pulses within the generated laser pulse train. In our experimental setup, we opted for a cube-shaped PBS where we rotated additionally the reflected light polarization plane by a half-wave plate (see Fig. S1a), to have it exactly at 90 deg with respect to the polarization plane of the transmitted light. After the 2nd PBS, a half-wave plate is used to change the polarization of both pulses to a diagonal one, which allows for their further splitting in the following DS. Nevertheless, as the polarization of beam reflected at PBS acquires also some ellipticity, it is advantageous to replace the half-wave plate by a diagonally oriented polarizer at least after a pair of DS, which purifies the linear polarization of all splitted laser pulses

and ensures the correct functionality of the following DS. By this approach, we were able to generate 16 laser pulses with very similar intensities that were co-propagating for several meters with nearly identical spatial profiles and having the selected mutual time spacing between them (see Fig. S1c).

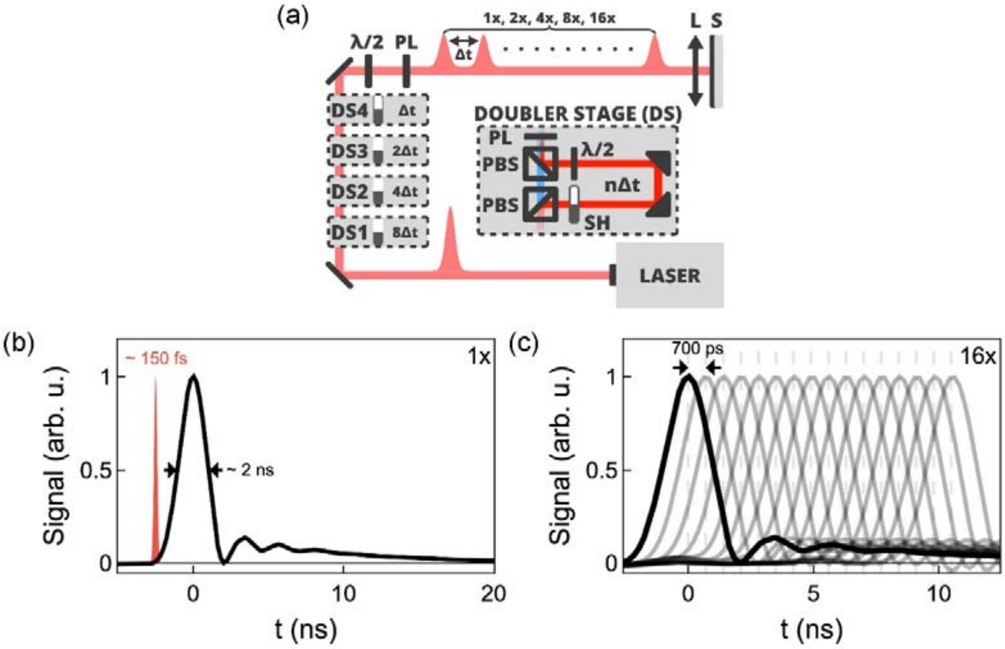

Supplementary Figure S1. Generation of a burst of laser pulses from a single laser pulse. a, Schematics of the corresponding experimental setup. A laser pulse is divided into up to 16 equiamplitude and equidistant Δt-delayed pulses by passing through doubler stages DS1-DS4, each of them inducing the indicated time delay, which form the pulse burst incident on the sample. Grey rectangle: An individual doubler stage (DS) where a diagonally polarized laser pulse is split and consequently spatially recombined by a pair of polarization beam splitters (PBS). Here, a delay line introduces a mutual time delay nΔt between the transmitted (blue path, horizontal polarization) and reflected pulse (red path, vertical polarization). A computer-controlled shutter (SH) allows to block the reflected pulse. A half-wave plate ($\lambda/2$) or a polarizer (PL) changes the polarization of both pulses to a diagonal one, allowing a serial concatenation of DS. **b**, Time-resolved signal measured by an avalanche photodiode (black) after impact of one 150-fs-long laser pulse (red). **c**, Signals measured for each of the 16 individual laser pulses from the burst, which are mutually time delayed for Δt = 700 ps.

Supplementary Note 2. Volume of the switched CuMnAs

The laser pulses were focused by lens on the device structure, producing a Gaussian intensity profile

$$I(r) = I_0 \cdot e^{-\frac{r^2}{w^2}} \tag{S2.1}$$

where r is the radial distance from the center of the Gaussian profile. The experimentally achieved beam size, characterized by a full-width $2w \approx 20$ µm, was determined using the knife-edge method. Due to the absorption in CuMnAs epilayer, the pulse intensity decays exponentially with an absorption coefficient of $\alpha = 3.1 \cdot 10^5$ cm^{-1} [S1]. The resulting energy distribution of the laser light (expressed as energy density per unit volume) within the epilayer is described by

$$U(r,z) = U_0 \cdot e^{-\frac{r^2}{w^2}} \cdot e^{-\alpha z}, \tag{S2.2}$$

where z denotes distance in the epilayer along the laser propagation direction, and U_0 is the amplitude of the energy density. The measured device resistance change ΔR is linearly proportional to the volume of the region in which the energy density in CuMnAs surpasses a threshold value, $U(r, z) \geq U_{TH}$. This volume is given by

$$V_{\mathrm{switched}} = \frac{\pi w^2}{2\alpha} \ln^2(\theta)\,;\ \theta \geq 1, \tag{S2.3}$$

where θ, defined as $\theta = U_0/U_{TH}$, measures the extent to which the threshold energy is exceeded. This quantity can also be represented in terms of the incident laser fluence F (energy density per unit area) as $\theta \approx F/F_{TH}$, where F_{TH} is the threshold fluence. If $\theta < 1$, the threshold condition for achieving the switching is not fulfilled, implying $V_{\mathrm{switched}} = 0$. This switched volume can be envisaged as a cap-like shape (see Fig. S2) with a circular base of diameter d:

$$d = 2w\sqrt{\ln(\theta)};\quad \theta \geq 1. \tag{S2.4}$$

Fig. S2b demonstrates how the diameter d expands as the switching threshold is exceeded. Despite the relatively large spot size of the incident laser pulse, a considerably smaller region can be switched.

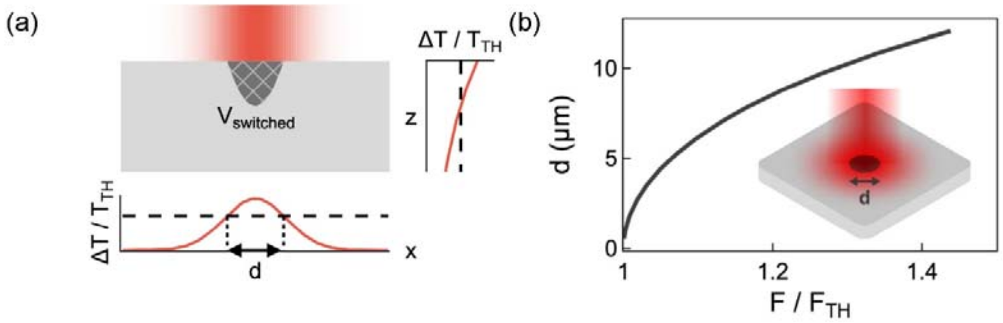

Supplementary Figure S2. Volume of CuMnAs with a resistance increased due to the quench-switching. a, The incident laser pulse with a Gaussian lateral (*x*) profile is absorbed in the CuMnAs epilayer with an exponentially decaying intensity profile in the *z*-direction. The volume $V_{switched}$ represents the region where the laser-pulse-induced temperature increase ΔT of CuMnAs exceeds the threshold temperature T_{TH}. This volume can be visualized as a cap-like structure with a circular base of diameter *d*. **b,** The dependence of *d* on fluence *F*, expressed relative to the value of the threshold fluence F_{TH}.

Supplementary Note 3. Measurement of heat dissipation speed from CuMnAs films to GaP substrate

The photoexcitation of a metal by an intense femtosecond laser pulse excites the electron distribution out of equilibrium on a time scale much shorter than the electron–phonon interaction time. The resulting non-thermal population of electrons thermalizes rapidly by electron–electron scattering processes. Consequently, a thermalized electron system, which can be described by a Fermi distribution with an electron temperature, is formed within ≈ 100 fs after the impact of the pump pulse. On a picosecond time scale, the excess energy is dissipated from the electron system to the lattice by electron–phonon scattering processes, which leads to an increase in the lattice temperature. Finally, heat diffusion dissipates the excess energy and the metal returns to the equilibrium state. Importantly, all the above effects lead to a change in optical properties and, therefore, the corresponding characteristic time constants can be evaluated from the measured optical transient signals [S1]. In Fig. S3 we show results of degenerate pump–probe experiment in in CuMnAs films of different thicknesses where a time evolution of pump-induced transmission change was measured by probe pulses of the same wavelength as that of the pump pulse. The lines are fits by a mono-exponential decay function with depicted characteristic time constants, which describe the heat dissipation from the CuMnAs epilayer to the GaP substrate. Clearly, the heat dissipation is much faster in thinner films.

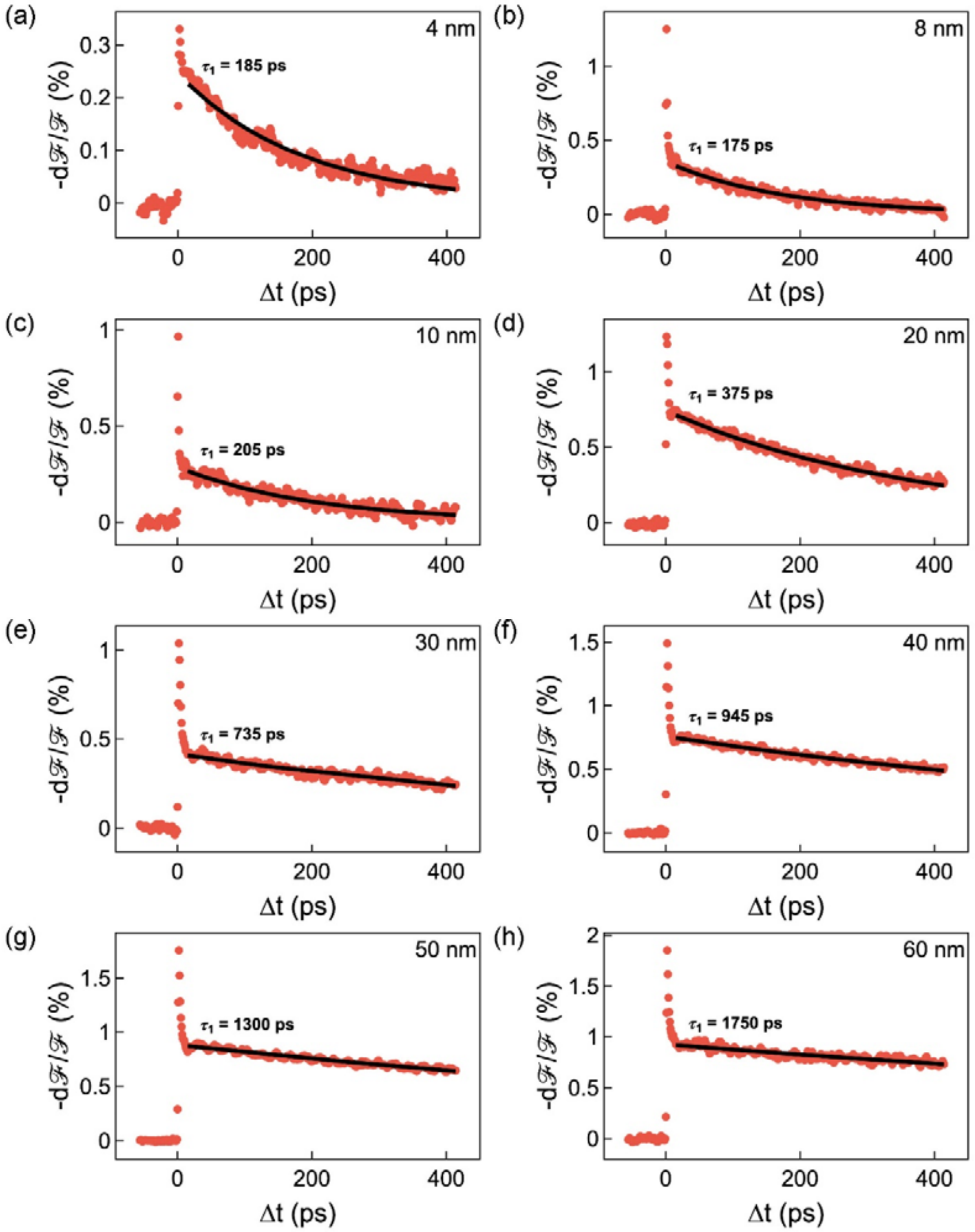

Supplementary Figure S3. Measurement of heat dissipation in CuMnAs films of different thicknesses. Laser-pulse-induced dynamics of transient decrease of differential transmission $d\mathcal{T}/\mathcal{T}$ measured at 15 K by optical pump-probe experiment in CuMnAs epilayers with indicated thicknesses **a-f**, using pump and probe pulses with a wavelength of 820 nm (dots) and pump fluence of 3 mJ.cm^{-2}. Lines are fits by a mono-exponential decay function with a characteristic time constant τ_1.

Supplementary Note 4. Dependence of STM dynamics on pulse spacing

The heat-based short-term memory (STM) functionality in our memory device is governed by an interplay between heat accumulation in CuMnAs film from successive femtosecond laser pulses and heat dissipation into the substrate and environment. Following the procedure described in detail in Ref. S3, we model the heat dynamics after absorption of a laser pulse by a tri-exponential relaxation comprising a film-thickness-dependent heat dissipation from the film to the substrate, which is characterized by a time constant τ_1, and two slower terms. These two terms, with time constants τ_2 and τ_3, are associated with a heat transfer from the substrate to the sample holder and environment, which do not depend on the film thickness. For the CuMnAs films used in our experiment, $\tau_1 \approx 375$ ps and 1 300 ps for the 20-nm-thick and 50-nm-thick films, respectively, (see Fig. S3) with a relative amplitude $A_1 = 0.67$. The slower components are ~10 ns and ~100 ns (see Fig. 2c in Ref. S3) with relative amplitudes $A_2 = 0.25$ and $A_3 = 0.08$, respectively. On the sub-microsecond timescales relevant to STM, all eventual slower dissipation channels to environment can be disregarded. However, this residual heat can be used for the long-term memory erasing, as discussed in Supplementary Note 5.

For a device excitation by multiple laser pulses with a fixed time spacing Δt, we assume a linear superposition of single-pulse thermal responses. The quench switching in CuMnAs occurs when the cumulative thermal response after n pulses $S_n(\Delta t)$ exceeds the threshold temperature T_{TH}. In our model, the relative value of T_{TH} and single-pulse heating amplitude A were calibrated using the 20-nm film experimental data shown in Fig. 3f of the main paper: for Δt = 700 ps, the switching onset occurs between 4 and 8 pulses. In Figs. S4a – S4c we show how the balance between the heat accumulation and dissipation is controlled by the mutual pulse spacing Δt for 20 nm and 50 nm CuMnAs films. When Δt = 100 ps (i.e. shorter than τ_1), 3 pulses are required to reach the threshold temperature for both film thicknesses, see Fig. S4a. When Δt = 5 000 ps (i.e. much longer than τ_1), 21 pulses are needed to achieve switching, again independently of the film thickness, see Fig. S4c. Only when Δt is comparable to τ_1, e.g. 700 ps as in Fig. S4b, the intended sensitivity of the number of pulses required for reaching the switching on the CuMnAs film-thickness is achieved. To put this analysis on a more quantitative basis, we plot in Fig. S4d the difference between the number of needed optical pulses (ΔNOP) in 50-nm and 20-nm-thick films. However, as this number is strongly sensitive to the exact choice of T_{TH} and A in our model calculations, this analysis was repeated for 1 000 uniformly sampled threshold temperatures within interval $[T_{TH} - A/2, T_{TH} + A/2]$, where A is the single-pulse induced heating.

The obtained values of ΔNOP are shown as points in Fig. S4d. From these values the average value $\langle \Delta NOP \rangle$ can be computed, which is depicted as a line in Fig. S4d. This dependence reaches a maximum at $\Delta t \approx 700$ ps, which is the time delay between pulses used to obtain the experimental results shown in Figs. 3 and 4 of the main paper.

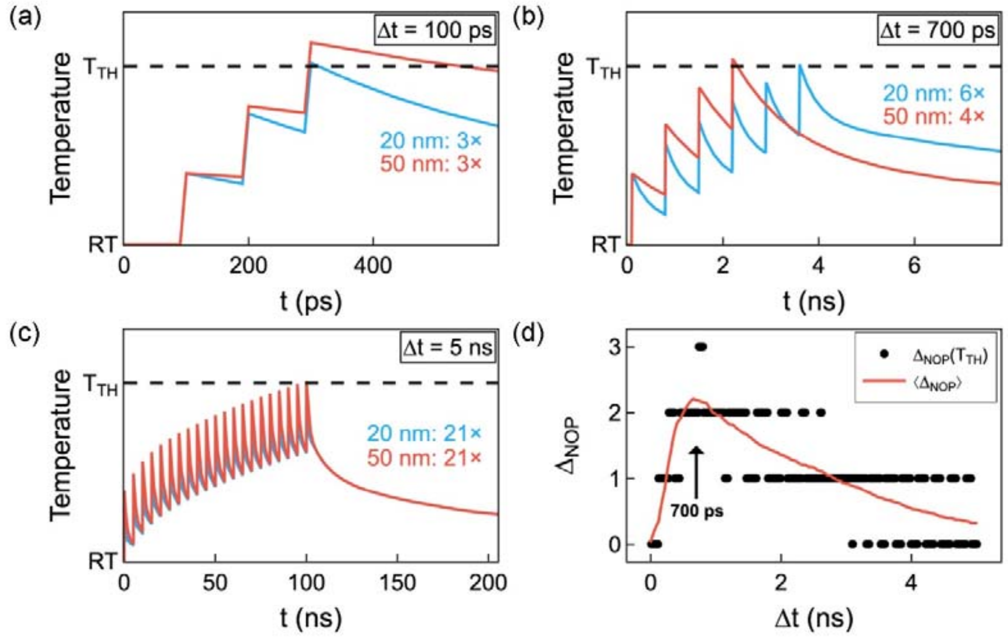

Supplementary Figure S4. Dependence of STM functionality on the pulse spacing between successive femtosecond laser pulses. a–**c**, Modeled cumulative temperature for 20-nm (blue) and 50-nm-thick (red) CuMnAs films exposed to bursts of laser pulses with different time spacing Δt: **a,** $\Delta t = 100$ ps, **b,** 700 ps, and **c,** 5 ns. Depicted numbers indicate how many laser pulses are needed for exceeding the threshold temperature T_{TH} for the corresponding film thickness. **d,** Difference between the number of needed optical pulses (NOP) for achieving the switching in 50-nm and 20-nm-thick films as a function of Δt (points), see text. Line is a threshold-averaged dependence that has a maximum around $\Delta t = 700$ ps, which was used to achieve the experimental results shown in Figs. 3 and 4 in the main paper.

Supplementary Note 5. Heat-based control of LTM erasing

As discussed in detail in the main paper, the incident laser pulses can lead not only to information memorizing (i.e., the increase of the device resistivity) but also to an acceleration of information forgetting, depending solely on the time spacing between the illuminating pulses. In this experiment, we used a 10 ns long memorizing period, when 16 rehearsals by laser pulses time-spaced by 700 ps were performed to achieve information transfer from the STM to the LTM. In Fig. 4d of the main paper, we illustrated that LTM forgetting can be significantly accelerated by laser pulses with the same intensity but with a time spacing of 1 ms. The reason why laser pulses with a time spacing of 700 ps cause the resistivity increase while the same pulses spaced by 1 ms cause the resistivity decrease lies in the non-commensurate time scales of the heat-related and quench-switching-related dynamics. If the pulse spacing is shorter than the time constants describing the main components of the heat-based dynamics of STM (i.e. (sub)nanosecond), the device excitation by a train of laser pulses leads to a temperature buildup, which induces the quench-switching in CuMnAs (see Fig. 3 in the main paper and Fig. S4). If the pulse spacing is longer (e.g., 1 ms), the accumulated temperature does not overpass the threshold temperature for the quench-switching but it leads to an acceleration of the strongly temperature-dependent relaxation rate from the high-resistive quench-switched states (see Figs. 4a and 4b in Ref. S2), as shown in Fig. 4d in the main paper. For even longer pulse spacing (above ~ 10 ms), also this effect disappears and the additional pulses have virtually no effect, as illustrated in Fig. S5a. To complete the picture, in Fig. S5b we show the results for the pulse spacing of 0.1 ms. Also in this case the accumulated temperature is not high enough to overpass the switching threshold and, consequently, the resistivity decrease is observed due to the laser-induced acceleration of the relaxation rate. Nevertheless, as the (quasi)equilibrium resistance of the metallic CuMnAs increases with the temperature (see Supplementary Fig. S4a in Ref. S3), the device resistance does not relax to the original equilibrium value but to a value corresponding to slightly heated CuMnAs.

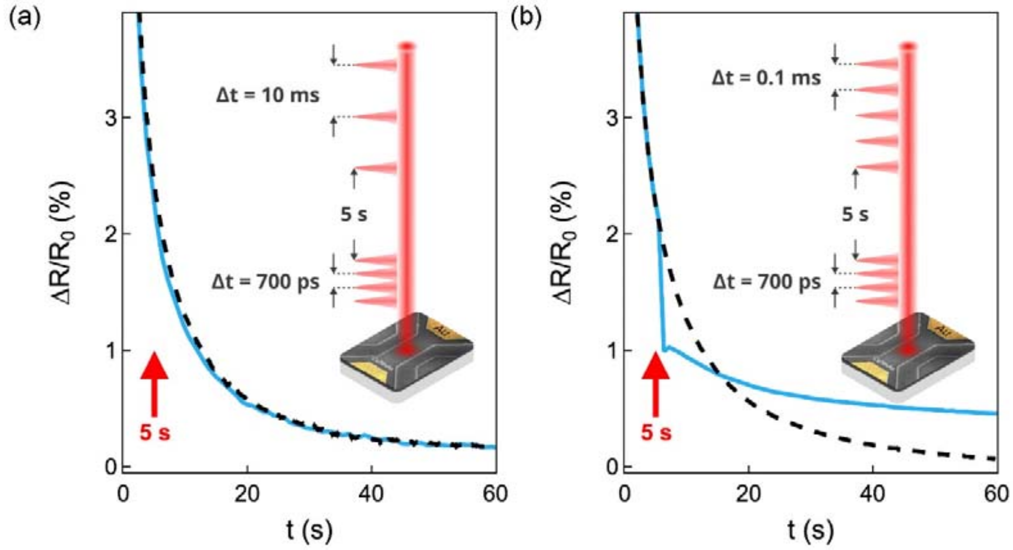

Supplementary Figure S5. Experimentally-measured dependence of laser-induced erasing of LTM on time spacing between the erasing pulses. The dashed curve shows the resistivity decay (i.e., a LTM "forgetting" dynamic) measured after a 10 ns long memorizing period, when 16 rehearsals by pulses with a fluence of 0.4 F_{TH} and a time spacing of 700 ps were used in a device fabricated from a 20-nm-thick film. The solid curve in **a,** and **b,** shows the forgetting dynamics when at 5 s laser pulses with the same fluence but with a time spacing of 10 ms and 0.1 ms, respectively, start to be incident on the memory device. The results for a time spacing of 1 ms are shown in Fig. 4d of the main paper.

To further clarify the boundaries between the write, erase, and inert regimes a simple case study of a temperature-induced acceleration of the post-switching CuMnAs relaxation was computed. see Fig. S6. As discussed in detail in Ref. S2, the decay of the high-resistive quench-switched state is described by a Kohlrausch (stretched-exponential) form with β = 0.6 and two, strongly temperature-dependent, relaxation times. At room temperature, these times are in the ~10 ms and ~10 s ranges [S2]. On the seconds-long time window relevant to Fig. 4d in the main paper and Supplementary Fig. S5, the fast component is already relaxed and the time evolution of the switching-induced decay of the resistance change can be modeled as

$$\frac{\Delta R}{R_0} \sim exp\left(-\left(\frac{t}{\tau_{QS}(T)}\right)^{0.6}\right). \tag{S5.1}$$

The temperature (T) dependence of the characteristic time constant τ_{QS} follows a simple exponential (Arrhenius) behavior

$$\tau_{QS}(T) = \tau_0 \, exp\left(\frac{E_{QS}}{k_B T}\right), \tag{S5.2}$$

where τ_0 is the attempt time, E_{QS} is the activation energy and k_B is the Boltzmann constant. Using τ_0 = 1 ps and E_{QS} = 0.8 eV [S2], we can reproduce the experimentally measured (a dashed line in Fig. S5) device resistance relaxation to the equilibrium value, as shown by the dashed line in Fig. S6. When the sample temperature is increased by the laser pulse train, which starts to be incident on the device 5 s after the memorizing period, τ_{QS} is reduced (see inset in Fig. S6b) and the resistance relaxation is accelerated, as shown in Fig. S6 for three values of the laser-induced temperature increase ΔT. Moreover, when ΔT exceeds ≈ 10 K, also the temperature dependence of CuMnAs resistence (Supplementary Fig. S4a in Ref. S3) has to be taken into account, as shown in Fig. S6c. Finally, we can compare the modelled resistance relaxations with those measured for different pulse spacing Δt. For Δt = 10 ms (Fig. S5a) the resistance relaxation is not visibly accelerated by the laser pulse train, implying ΔT < 1 K (Fig. S6a). For Δt = 1 ms (Fig. 4d in the main paper) and 0.1 ms (Fig. S6b) the laser-induced temperature increase seems to be around 5 K (Fig. S6b) and 15 K (Fig. S6c), respectively. This shows that the characteristic time constant of the heat dissipation, which is responsible for this laser-induced erasing of LTM due to the sample temperature build-up (analogous to Fig. S4), is in the milisecond time range.

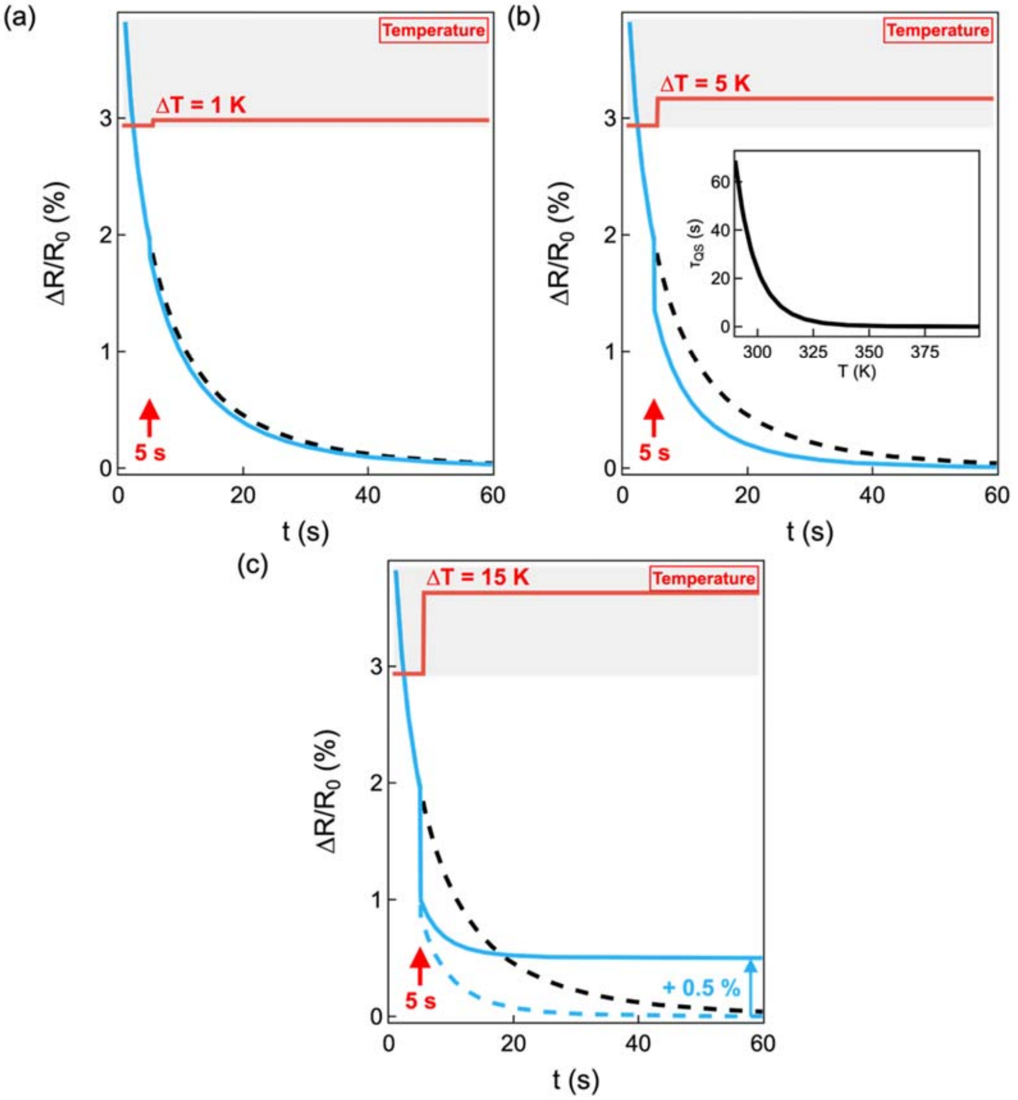

Supplementary Figure S6. Modelling of temperature-induced erasing of LTM. Black dashed lines are the room temperature resistance relaxation following the information writing in LTM, as modelled by Eq. (S5.1). When the CuMnAs temperature is increased at $t = 5$ s by a train of laser pulses (see the upper parts of the figures), the time constant τ_{QS} is reduced, as shown in the inset in **b**. **a**-**c**, Resistance dynamics (blue lines) obtained for depicted temperature profiles with ΔT = 1 K, 5 K, and 15 K, respectively. In **c**, due to a considerable temperature increase of 15 K, the resistance does not relax to the equilibrium value (blue dotted line) but to a resistance increased by ≈ 0.5% (see the temperature dependence of R_0 in Supplementary Fig. S4a in Ref. S3).

References

[S1] Surýnek, M., Saidl, V., Novák, V., Campion, R.P., Wadley, P., & Němec, P. Investigation of magnetic anisotropy and heat dissipation in thin films of compensated antiferromagnet CuMnAs by pump–probe experiment. *J. Appl. Phys.* **127**, 233904 (2020).

[S2] Kašpar, Z. *et al.* Quenching of an antiferromagnet into high resistivity states using electrical or ultrashort optical pulses. *Nature Electronics* **4**, 30–37 (2021).

[S3] Surýnek, M. *et al.* Picosecond transfer from short-term to long-term memory in analog antiferromagnetic memory device. Newton **1**, 100034 (2025).
